# Ultrahigh β-phase content poly(vinylidene fluoride) with relaxor-like ferroelectricity for high energy density capacitors

**DOI:** 10.1038/s41467-019-12391-3

**Published:** 2019-10-18

**Authors:** Nan Meng, Xintong Ren, Giovanni Santagiuliana, Leonardo Ventura, Han Zhang, Jiyue Wu, Haixue Yan, Michael J Reece, Emiliano Bilotti

**Affiliations:** 1 School of Engineering and Materials Science, Queen Mary University of London, Mile End Road, London, E1 4NS UK; 2 NPU-QMUL Joint Research Institute of Advanced Materials and Structures, Queen Mary University of London, Mile End Road, London, E1 4NS UK

**Keywords:** Energy science and technology, Materials for energy and catalysis

## Abstract

Poly(vinylidene fluoride)-based dielectric materials are prospective candidates for high power density electric storage applications because of their ferroelectric nature, high dielectric breakdown strength and superior processability. However, obtaining a polar phase with relaxor-like behavior in poly(vinylidene fluoride), as required for high energy storage density, is a major challenge. To date, this has been achieved using complex and expensive synthesis of copolymers and terpolymers or via irradiation with high-energy electron-beam or γ-ray radiations. Herein, a facile process of pressing-and-folding is proposed to produce β-poly(vinylidene fluoride) (β-phase content: ~98%) with relaxor-like behavior observed in poly(vinylidene fluoride) with high molecular weight > 534 kg mol^−1^, without the need of any hazardous gases, solvents, electrical or chemical treatments. An ultra-high energy density (35 J cm^−3^) with a high efficiency (74%) is achieved in a pressed-and-folded poly(vinylidene fluoride) (670-700 kg mol^−1^), which is higher than that of other reported polymer-based dielectric capacitors to the best of our knowledge.

## Abstract (web summary)

Dielectric materials are candidates for electric high power density energy storage applications, but fabrication is challenging. Here the authors report a pressing-and-folding processing of a dielectric with relaxor-like behavior, leading to high energy density in a polymer-based dielectric capacitor.

## Introduction

The demands of reducing both CO_2_ emissions and the consumption of fossil fuels require an enhancement of the efficiency of energy usage and the long-term pursuit of renewable and sustainable energy sources (such as solar, wind, wave, etc.). These energy sources are intermittent, and it is therefore of paramount importance to develop efficient, low-cost and environmentally friendly electric energy storage systems. Currently, there are three main options: batteries, electrochemical capacitors and dielectric capacitors^1^. Batteries, with their high energy density (lead-acid battery: 200–400 J cm^−3^ and lithium ion: 900–2500 J cm^−3^) and low power density (<500 W kg^−1^), are usually used in applications that require relatively slow release of energy (>100 s), while capacitors, with their high power density (electrochemical capacitors: 10^1^–10^6^ W kg^−1^ and dielectric capacitors: ~10^8^ W kg^−1^), are used for rapid release of energy (<0.01 s)^2^. Electrochemical capacitors have at least an order of magnitude higher energy density compared to dielectric capacitors, but suffer from lower power density and lower output voltage. Only dielectric capacitors can meet the requirement of ultrahigh power density (up to ~10^8^ W kg^−1^)^1^, with the added benefits of long service life and fast release of energy^3^, which makes them important for high power/pulse power technologies, such as motor drives, mobile power systems, space‐vehicle power systems, electrochemical guns, and so on^4,5^.

However, the application of dielectric capacitors is currently limited by their low energy density, especially in applications requiring large capacitances and small packaging size. For example, the best-performing commercial dielectric capacitor, biaxially oriented polypropylene (BOPP), only has an energy density of ~1–2 J cm^−3^, which is an order of magnitude lower compared to commercial electrochemical capacitors (20–29 J cm^−3^)^6,7^. Therefore, achieving high energy density in dielectric capacitors is a major bottleneck in extending their practical applications. The recoverable energy storage (*U*_rec_) for dielectric capacitors is generally described by the following equation: *U*_rec_ = ∫*E*d*D*, where *E* and *D* are electric field and displacement, respectively. Specifically, for linear dielectrics, *U*_rec_ = $\mathop {\smallint }\nolimits^ E{\mathrm{d}}D = \frac{1}{2}DE = \frac{1}{2}\varepsilon _0\varepsilon _{\mathrm{r}}E_{\mathrm{b}}^2$, where *ε*_0_ and *ε*_r_ are the vacuum and relative dielectric constant, respectively, and *E*_b_ is the breakdown field. *U*_rec_ has a quadratic dependence on *E*_b_. While polymers possess much lower dielectric constants compared to inorganic materials, they have at least an order of magnitude higher energy storage capacity as a result of their much higher breakdown fields (several hundreds of kV mm^−1^). Apart from their high *E*_b_, polymers have additional advantages, such as low density, high processability, mechanical flexibility and high toughness.

The relatively low *ε*_r_ of polymers is the limiting factor for their *U*_rec_. The above-mentioned BOPP, for instance, has a *ε*_r_ of ~2. Polar polymers, with dipole moments in their polymer chains, can exhibit much higher *ε*_r_. Poly(vinylidene fluoride) (PVDF) is a typical example^8–10^ with a *ε*_r_ of ~10. PVDF has at least four well-defined phases, α-, β-, γ- and δ-phase. The α-phase is non-polar, while the other three are polar phases, of which the β-phase shows the highest polarization and the most favourable ferroelectric properties^11–14^. However, PVDF crystallizes predominantly into α-phase from the melt, with fairly low content of β-phase (<8%)^15^, which can be increased by solid-state drawing and/or high electric field poling (~50–85%)^16^. β-PVDF exhibits broad ferroelectric hysteresis loops and is not suitable for energy storage (Supplementary Fig. 1)^17^. Relaxor ferroelectric (RFE) or anti-ferroelectric (AFE) behaviour is instead desirable for energy storage (Supplementary Fig. 1)^17^. However, RFE or AFE behaviour have only been reported in the case of electron- or γ-irradiated poly(vinylidene fluoride-trifluoroethylene) P(VDF-TrFE)^18^, ternary polymers poly(vinylidene fluoride-trifluoroethylene-chlorofluoroethylene) P(VDF-TrFE-CFE)^19^ and poly(vinylidene fluoride-trifluoroethylene-chlorotrifluoroethylene P(VDF-TrFE-CTFE)^20^ and PVDF-based graft polymers such as P(VDF-CTFE)-*graft*-polystyrene^21^. The complexity and cost of the above polymers and processes present a major barrier to their commercial use.

Our work focuses on virgin, commercially available and inexpensive PVDF homopolymers, and demonstrates a facile and scalable processing route to obtain an ultrahigh content of β-phase (~98% of crystalline phase) with RFE-like behaviour that has an exceptionally high energy storage density of 35 J cm^−3^, which is achieved by reversible field-induced transitions related to thermally unstable local polar structures. This is the highest value reported for a polymer-based dielectric material (Supplementary Table 1)^22–31^, to the best of our knowledge. The produced homopolymer PVDF films show the lowest dielectric loss (0.02 at 1 kHz) and highest maximum working temperature (120 °C) in PVDF-based ferroelectric materials (dielectric loss: 0.03–0.05 for previously reported PVDF and copolymer films; 0.08–0.10 for terpolymer films; maximum working temperatures: 100–120 °C for previously reported PVDF and copolymer films; 50–60 °C for terpolymer films), which challenges the performance of commercial electrochemical capacitors and ceramic capacitors, with the added benefits of mechanical flexibility, toughness and low density.

## Results

### Pressed-and-folded PVDF for electric energy storage

**Fig. 1:**
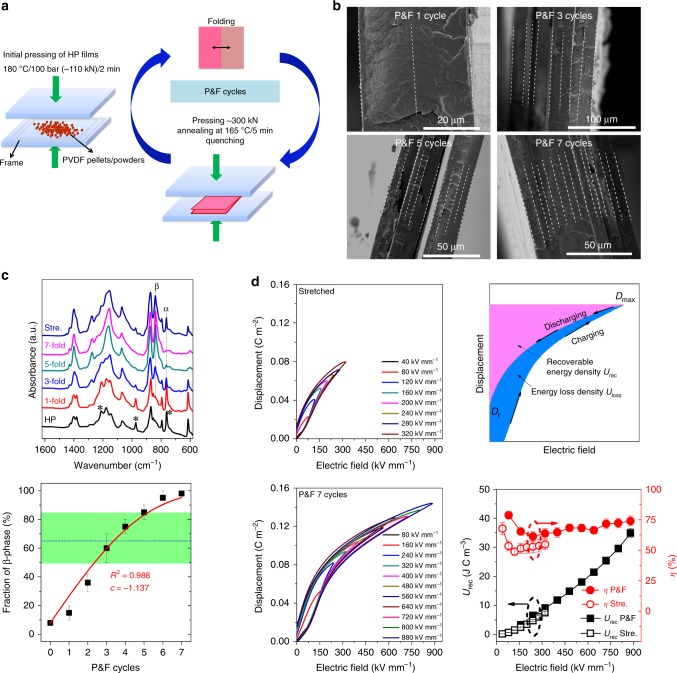
Pressed and folded poly(vinylidene fluoride) compared with stretched samples. **a** Schematic demonstration of P&F technique. **b** Cross-sectional SEM images of P&F samples folded at 165 °C and 300 kN after different numbers of folding cycles. A fine and discrete layered structure is generated during P&F. **c** The evolution of crystalline phases revealed by the FTIR absorbance spectrum. The initial hot-pressed (HP) films mainly crystallized into α-phase with characteristic peaks at 764, 975 and 1212 cm^−1^ highlighted by asterisks and transformed to about 98% β-phase (strong peak at 840 cm^−1^) after seven folding cycles. The horizontal green shaded area indicates the reported values of fraction of β-phase in commonly stretched PVDF films, the blue dashed line represents the fraction of β-phase for the stretched film in this work and the continuous line is the best fit of the experimental P&F data with Eq. 2 using *c* as free parameter (*f*_β0_ and *h* were set at 8% and 0.6, respectively). **d** A comparison of electric energy storage properties of P&F and stretched films, which includes unipolar ferroelectric hysteresis loops, schematic calculations of stored energy of ferroelectric materials and the recoverable energy density *U*_rec_ and energy efficiency *η* of P&F and stretched films

Our approach uses a unique processing route called “pressing-and-folding” (P&F), which draws inspiration from the process used by bakers to prepare puff pastry and croissants^32^. P&F is an iterative process in which the P&F cycle can be applied an arbitrary number of times (Fig. 1a). Each P&F cycle is composed of a folding step, in which an approximately rectangular PVDF film, produced by hot pressing (HP), is folded, followed by a pressing and annealing step around the melting point (*T*_m_) of PVDF (160–170 °C). For example (samples characterized in Fig. 1b), the initial films (~50 mm × 50 mm, thickness ~200 μm) were first folded and cold pressed with a compression force of 300 kN. The temperature was then increased from ambient temperature to 165 °C. The folded films were then held for 5 min under the conditions of 165 °C and 300 kN, followed by cold water quenching while maintaining the same compressive force. The maintenance of pressure during cooling was observed to facilitate the formation of β-phase (Supplementary Fig. 2). During P&F, a fine and discrete layered structure is generated, with the thickness of each layer decreasing with the number of P&F cycles (Fig. 1b). As expected, the initial HP films (no folding) contained predominantly α-phase, as confirmed by Fourier transform infrared (FTIR) spectroscopy (presence of characteristic peaks of α-phase at 764, 975 and 1212 cm^−1^, absence of the β/γ characteristic peaks at 840 cm^−1^) (Fig. 1c)^33,34^. Surprisingly, P&F produced films containing about 98% of β-phase after seven P&F cycles (Fig. 1c), higher than for any other reported method (max 85% by unidirectional/biaxial drawing, as highlighted by the horizontal green shaded area in Fig. 1c)^16,35–38^. The analysis of PVDF phase content has been well studied in previous research works^36–38^, which includes the determination of existing phases according to the appearance of exclusively characteristic bands (e.g. α-phase: 764 cm^−1^; β-phase: 1275 cm^−1^ and γ-phase: 1234 cm^−1^ listed in Supplementary Table 2) and then calculation using specific equations based on the sample containing α- and β- phase only or containing a mixture of α-, β- and γ-phase. More details are summarized in Supplementary Note 1. The folded samples show similar melting points compared to the initial HP materials (no folding) (Supplementary Fig. 3 and Supplementary Table 3). The densities of the films were measured based on the Archimedes principle, and are ~1.770 and 1.780 g cm^−3^ for HP and P&F samples, respectively. Consequently, the degree of crystallinity of HP and P&F samples are 41% and 38%, respectively, which was calculated using the densities of amorphous PVDF, pure α- and pure β-PVDF of 1.68, 1.92 and 1.97 g cm^−3^, respectively^39,40^. The calculated values of the degree of crystallinity are typical of PVDF^41^. For comparison, HP films were solid-state drawn to failure at 100 °C and 10 mm min^−1^, which was reported to be the optimum conditions for obtaining the β-phase in a previous work^35^, achieving only ~65% of β-phase content (indicated by the blue dashed line in Fig. 1c) and a degree of crystallinity of 36% (density: 1.772 g cm^−3^). The ferroelectric hysteresis loops of the stretched films (thickness ~35 μm) could only be tested at relatively low electric fields (<350 kV mm^−1^) due to the max limit of 10 kV applied voltage of our equipment (Fig. 1d). From the schematic diagram of stored energy in ferroelectrics (Fig. 1d), a lower remnant displacement (*D*_r_), a higher maximum displacement (*D*_max_) and a higher breakdown field (*E*_b_) than the values found for the stretched films^31^ would be favourable to obtain higher recoverable energy density *U*_rec_. The P&F sample (one layer extracted from a film processed with seven P&F cycles, as described in the section Materials and film preparation in Methods) showed an extremely high *E*_b_, 880 kV mm^−1^. The *D*_r_ and *D*_max_ are 0.017 and 0.144 C m^−2^, respectively. All of these parameters contribute to a high *U*_rec_ of 35 J cm^−3^, which is the highest value ever reported for any polymer-based dielectric capacitor. Despite the ultrahigh *U*_rec_, the P&F samples also had higher energy efficiency *η* (~74%) compared to the stretched film (~54%) (Fig. 1d). Moreover, the P&F films showed high breakdown strength with an average value of 789.5 kV mm^−1^, as evaluated according to the criterion of Weibull distribution statistics (Supplementary Fig. 4).

The above results raise two fundamental questions: Why does P&F generate such a high content of β-phase (~98%)? and Why do P&F films show relaxor-like ferroelectric behaviour?

### Phase transformation from α to β during the P&F process

**Fig. 2:**
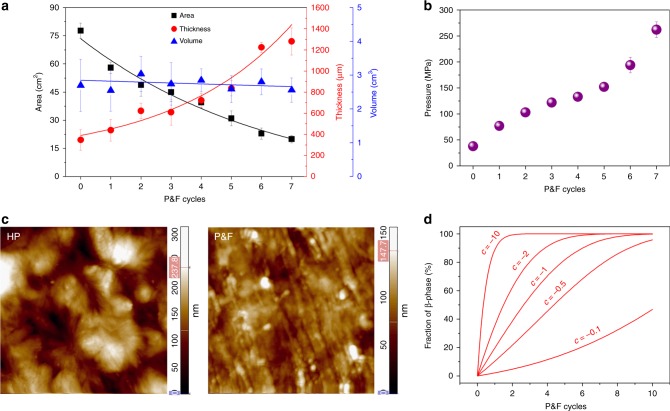
Morphological changes of poly(vinylidene fluoride) samples. **a** Variation of overall sample area, thickness and volume with the number of P&F cycles. **b** Calculated pressure (nominal stress) in purple data points for different P&F cycles. **c** AFM topography images (5 µm × 5 µm) of initial HP (left) and P&F (right) after seven cycles, which show the evolution of morphology, from large spherulites in HP films to small granules after P&F films. **d** Theoretical changes of the β-phase content according to Eq. 2 with different values of parameter *c*

To answer the first question, we have investigated the dimensional changes of a sample during the P&F process (Fig. 2a). The sample deformed plastically at each cycle, but its volume always remained constant according to the experimental results (Fig. 2a). The maximum nominal stress (or pressure, Fig. 2b) reached at the end of each cycle increases during the process as $P_i\left( n \right) = P_1\left( {\frac{2}{a}} \right)^{n - 1}$, where *n* is the number of cycles, *P*_1_ is the pressure applied at the first cycle and *a* = 1.65 (±0.14) is a factor that describes how much the sample area increases during compression (Supplementary Note 2). Atomic force microscopy (AFM) images (Fig. 2c) reveal the change of morphology during P&F, from spherulites (~2–3 μm in diameter) in the initial HP film to small granular structure (~150 nm in diameter) due to the increasing applied pressure with increasing number of P&F cycles.

The energy (*Ω*) that plastically deforms a unit volume of polymer provides the “driving force” for the phase transformation, and it grows with the number of cycles as $\Omega \left( n \right) = P_1\left( {1 - h} \right)\frac{{1 - \left( {2h} \right)^n}}{{1 - 2h}}$ (Supplementary Note 2), where *h* = 0.61 (±0.09) is a factor that describes how much the sample thickness decreases during compression. Within this context, it is worth noting that the β-phase has a smaller unit cell volume (108.2 Å^3^) compared to the α-phase (118.6 Å^3^) for the same number of atoms^11^. Moreover, judging by the calculated values of intra- and inter-molecular potential energy, −6.03 and −5.73 kcal mol^−1^ for α- and β-phase, respectively, the β-phase is slightly energetically less stable^42^. The imposition of the ever increasing pressure *P*_i_ leads to a closer packing of the atoms and, as a result, a favoured β-phase with all-*trans* chain conformation. These considerations are supported by studies on phase transformation in solid-state drawn^43,44^, solid-state compression^45^ and rolling processed^46^ PVDF films. The amount of β-phase (*f*_β_) is proportional to *Ω*. Therefore, an advantage of the P&F process over the drawing process is that PVDF can always adsorb more “plastic energy” in compression than in tension^47^. Compression can be carried out in a broader (lower) temperature range than tension. Overall, the P&F process can allow higher phase conversions. Summarizing, the increment of β-phase (Δ*f*_β_) depends on the increment of plastic energy (Δ*Ω*) adsorbed by the material, and must be limited by the amount of α-phase, that is, by the term 1 – *f*_β_, as when all of the α-phase is converted there cannot be further increments of β-phase. From these considerations, and assuming that the fraction of β-phase before starting the P&F process is *f*_β0_ (8%), a differential calculation leads us to the following relationship between the amount of β-phase and plastic energy:

1 $$f_{\upbeta} \left( \Omega \right) = 1 - \left( {1 - f_{\upbeta 0}} \right)e^{ - b\Omega }$$

or, in terms of P&F cycles,

2 $$f_{{\upbeta}} \left( n \right) = 1 - \left( {1 - f_{{{\upbeta}}0}} \right)e^{ - c\left[ {1 - \left( {2h} \right)^n} \right]},$$

where *b* and $c = bP_1\frac{{1 - h}}{{1 - 2h}}$ are some proportionality constants. Figure 1c shows that the experimental data of *f*_β_ can be well fitted by Eq. 2 with *c* = −1.1, whereas the effects of different values of *c* on the β-phase amount during the P&F process is illustrated in Fig. 2d. A number of factors should influence the relationship between *f*_β_ and *Ω* (i.e. the proportionality constant *b* or *c*), as well as the amount of *Ω* that can be generated in the P&F process. We have tried to identify some of these factors, as it is clearly desirable to transform as much α-phase into β-phase as is possible with the lowest amount of plastic energy, or number of cycles.

**Fig. 3:**
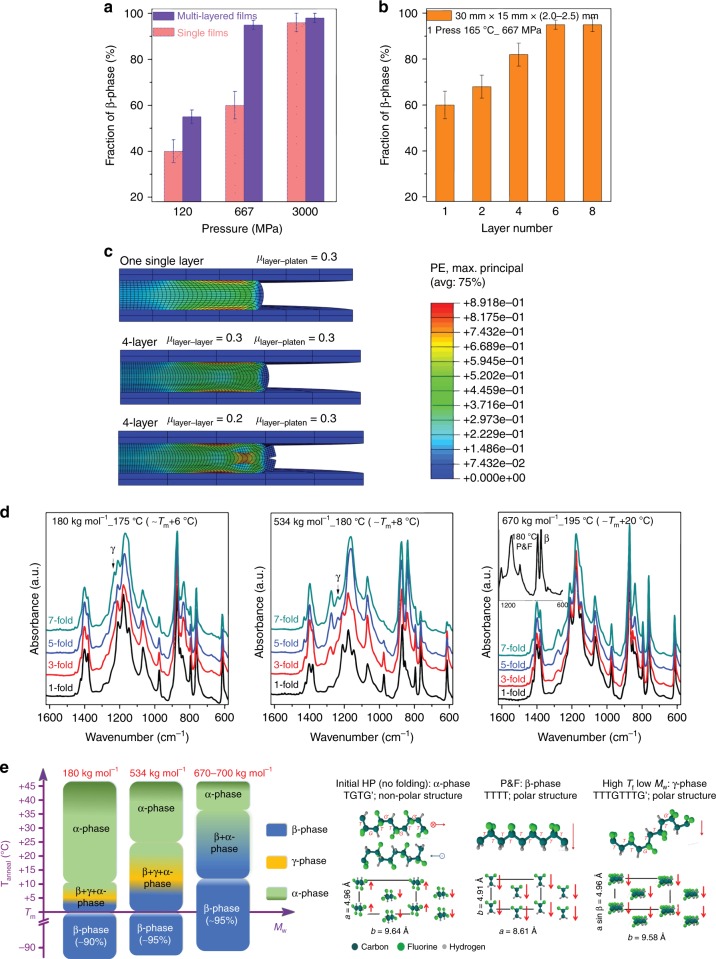
Factors influencing the formation of β-phase during processing. **a** Comparison of the phase transition in samples (670–700 kg mol^−1^) with monolayer and six multi-stacked layers at three different pressures (force 300 kN), 120, 667 and 3000 MPa, followed by pressure annealing at 165 °C for 5 min, which indicates that high pressure and layered structure favour the formation of β-phase. **b** Fraction of β-phase for samples (670–700 kg mol^−1^) with the same dimensions, but composed of different numbers of layers that were pressed once at 667 MPa (force 300 kN) followed by pressure annealing at 165 °C for 5 min and cold water quenching. **c** FEM simulations of film compression in the cases of monolayer film, four-layer film with same friction coefficients *µ* between layer and platen and between layers, and four-layer film with *µ* between layer and platen higher than *µ* between the layers. The bar scale represents the overall strain deformations. **d** FTIR of samples with different *M*_w_, which indicates the formation of γ-phase in PVDF (low *M*_w_) at higher *T*_anneal_ (denoted by the γ-characteristic band at 1234 cm^−1^) and high *M*_w_ favours the transformation of β-phase (inset). For PVDF with *M*_w_ of 180, 534 and 670 kg mol^−1^, *T*_m_ was 169, 172 and 172 °C, respectively. **e** Demonstration of phase evolution with *T*_anneal_ and *M*_w_ during P&F and schematic diagram of chain conformation, crystal structure and polarization of PVDF. The HP (no folding) films are mainly α-phase, with two *trans*-gauche-trans-gauche′ (TGTG′) chains anti-parallel packed in a pseudo-orthorhombic unit cell. The symbols and arrows represent the in-plane and out-of-plane contributions to the dipole moments. After P&F at different temperatures (room temperature to *T*_m_), films formed into β-phase (>90 wt %) with all-*trans* (TTT) chain conformation. Low *T*_anneal_ and high *M*_w_ favour the formation of β-phase

First of all, the compressive stress–strain curve of PVDF is strain-rate-dependent^48^. Therefore, for the same final nominal stress, the energy adsorbed depends on the final strain; hence, there may be quicker phase conversion rates when pressing at higher strain rates. The second factor is the initial area (*A*_0_) of the sample, a smaller *A*_0_ corresponds to higher *P*_1_, hence greater *Ω*. If *A*_0_ is small enough, the pressure applied to the sample could be big enough to induce total phase transformation in fewer cycles. Figure 3a shows the β-phase content of single-layer films (left-hand dataset) having different *A*_0_ (25, 4.5 and 1 cm^3^) after the initial pressure *P*_1_; the film with the smallest *A*_0_ allowed almost a complete phase transformation (95 ± 5%) in just one cycle. Figure 3a also shows the importance of a third factor, the number of initial stacked layers constituting a film. At the same *P*_1_, films made with six layers of HP films (Fig. 3b) presented much higher β-phase amounts than single-layer films. An explanation for this could lie in the friction between the PVDF and the hot-press platens. The friction induces shear stresses that could dissipate energy^48^ and reduce the pure elongation of the material in the plane perpendicular to the compression direction. Finite element method (FEM) simulations of film compression (Fig. 3c) confirm that the internal layers of a multilayer structure can elongate and plastically deform more than the external layers, especially when the friction between the layers is smaller than the friction with the platens.

The two last important factors are the temperature of the annealing step (*T*_anneal_) that follows the full closure of the hot press and the molecular weight (*M*_w_) of PVDF. They have a similar effect: lower *T*_anneal_ and higher *M*_w_ give lower chain mobility. Figure 3d presents the FTIR spectra of different *M*_w_ films annealed above their *T*_m_; the β-phase increased more remarkably during the P&F process for higher *M*_w_ (>530 kg mol^−1^) PVDFs. Moreover, in the low *M*_w_ (180 kg mol^−1^) films also some γ-phase (chain conformation T_3_GT_3_G′; Fig. 3e) was produced. This is consistent with a previous work reporting α- to γ-phase transition in PVDF (*M*_w_ 180 kg mol^−1^) under a minimal applied pressure of 200 kPa and a temperature range of 167–180 °C^49^. Apart from the *M*_w_, the *T*_anneal_ plays a critical role; regardless of their *M*_w_, all of the films presented only β-phase (>90%) when processed below their *T*_m_, but maintained the non-polar α-phase when *T*_anneal_ >> *T*_m_ (Fig. 3e and Supplementary Fig. 5). More details on the factors influencing the phase transformation can be found in the Supplementary Note 2.

### Relaxor-like ferroelectric behaviour in P&F films

**Fig. 4:**
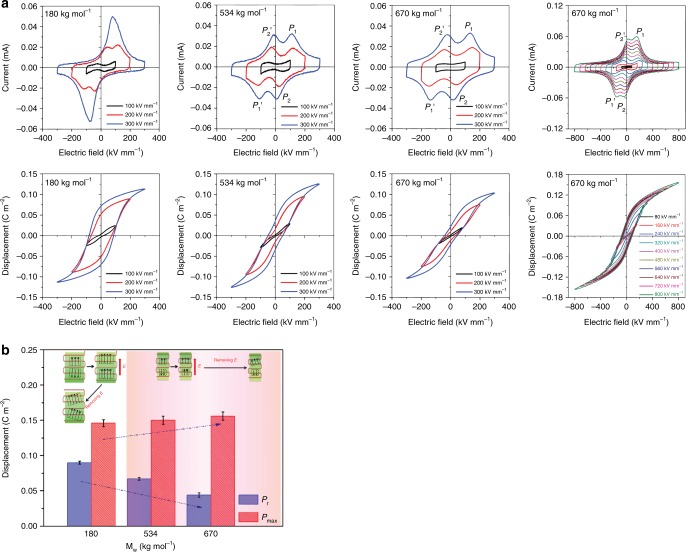
Ferroelectric properties of pressed and folded poly(vinylidene fluoride) samples. **a** Bipolar current–electric field (*I–E*) and displacement–electric field (*P–E*) loops of P&F PVDF after seven cycles with different *M*_w_ of 180, 534 and 670 kg mol^−1^. *P*_1_ and *P*_1_′ denote the peaks appeared during the application of electric field. *P*_2_ and *P*_2_′ denote the peaks appeared during the reversal of electric field. **b** A comparison of bipolar remnant polarization *P*_r_ and maximum polarization *P*_max_ of P&F films with different *M*_w_, and schematic diagram of polar structure change during charging and discharging processes, where the green blocks represent the crystallites, red lines represent the polymer chains with arrows denoting the dipole moment and the dashed lines highlight the polar structure, which was enhanced and grew larger under electric field

Having observed the formation of ultrahigh content of β-phase by P&F and proposed a possible mechanism, hereafter we focus on the ferroelectric behaviour of P&F PVDF films. The bipolar ferroelectric current–displacement–electric field (*I*–*D*–*E*) characteristics were measured under different electric fields at room temperature using a triangle waveform with a frequency of 10 Hz (Fig. 4). It is noted that the *D*–*E* loops alone are insufficient to demonstrate ferroelectric properties and clarify the type of ferroelectric behaviour. Instead, it is more informative to study the raw *I*–*E* curves, in which the presence of ferroelectric domain switching peaks and/or field-induced structure transition can easily be identified. Only some of the P&F samples with higher *M*_w_ polymer samples (534 and 670 kg mol^−1^) showed relaxor-like ferroelectric behaviour^50^ (Fig. 4a), that is, slim *D*–*E* loops with low *D*_r_ and high *D*_max_. The *M*_w_ 180 kg mol^−1^ polymer samples showed four current peaks located in the first and third quadrant at electric fields lower than 200 kV mm^−1^, which merged into two current peaks at 300 kV mm^−1^ (Fig. 4a), indicating the field-induced polar structural changes in P&F samples with low *M*_w_ (180 kg mol^−1^). This is further proved by measuring the second ferroelectric hysteresis loops (Supplementary Fig. 6), which differ from those obtained on pristine films (Fig. 4a), and demonstrate that the above structural changes are not reversible. The higher *M*_w_ P&F samples (534 and 670 kg mol^−1^) show four current peaks located in all of the four quadrants (denoted as *P*_1_, *P*_1_′, *P*_2_ and *P*_2_′ in Fig. 4a), which is related to the reversible field-induced polar structural changes. *P*_1_ and *P*_1_′ correspond to the regulation of polar structures upon the application of electric field, and the appearance of *P*_2_ and *P*_2_′ demonstrates that the polar structures can reverse back to the original state upon the removal of electric field, which is also proved by almost the same shape of re-measured hysteresis *I*–*D*–*E* loops (Supplementary Fig. 6). Note that *P*_2_ and *P*_2_′ shifted across the *y*-axis with further increase in the electric field up to 800 kV mm^−1^, which makes the four current peaks locate in the first and third quadrant and highlights the existence of partial irreversible phase transition due to the influence of ultrahigh electric field.

Previous to P&F, PVDF was reported to show double hysteresis loops, but only at an extreme condition^51^, −100 °C, 0.003 Hz and 240 kV mm^−1^, and more importantly, detailed studies with regard to *I*–*E* loops were not presented. Zhu et al.^52^ also studied the ferroelectric hysteresis loops of e-beam-irradiated PVDF-TrFE- and PVDF-TrFE-based terpolymers, and concluded that pinning polymer chains makes the e-beam-irradiated PVDF-TrFE show RFE narrow hysteresis loops and PVDF-TrFE-based terpolymers present double hysteresis loops; however, without the interpretation of *I*–*E* curves, the underlying physical mechanisms cannot be precisely decoded. The double hysteresis *D*–*E* loops can be produced by the phase transition between anti-ferroelectric to ferroelectric^53^ and paraelectric to ferroelectric^54^ with two completely different physics mechanisms. In the literature, PVDF-TrFE with high content of TrFE (47/53 and 37/63 mol%) and PTrFE materials were reported to show four current peaks located in all four quadrants^55,56^ (<150 kV mm^−1^), which is ascribed to the reversible field-induced structural change between the disordered *trans* phase (weak polar) and well-ordered all-*trans* phase (strong polar), as supported by in situ X-ray diffraction (XRD) measurement with applied electric field, being consistent with our analysis for P&F PVDF.

XRD was conducted to gain further insight into the field-induced structural changes in the P&F samples (Supplementary Fig. 7). The XRD data show a reduction of the mean size of the crystallites, from ~20 nm in the initial HP films to ~5 nm after the P&F treatment (Supplementary Table 4). The average size of the crystallites in the P&F samples decreased from 6 to 3.5 nm with increasing *M*_w_ from 180 to 670–700 kg mol^−1^ (Supplementary Table 4), which can be related to the reduced chain mobility with increasing *M*_w_. In P&F films with low *M*_w_, it is believed that relatively large-sized polar structures possess higher thermal stability^57^. The application of an external electric field will induce the re-orientation of CF_2_ dipoles and cause the regularization of the polymer chains^58^, leading to a structural change of long-range ferroelectric order (schematic images in Fig. 4b). In the P&F films with high *M*_w_ (534 and 670–700 kg mol^−1^), the small-sized polar structures are not stable due to the randomization of polarization induced by thermal activation^57^. In this case, the polar structures remain relatively small, despite the influence of the external field (schematic images in Fig. 4b). Consequently, a significant fraction of dipoles will reverse upon decreasing the electric field, as evidenced by the much lower *D*_r_, 0.020 C m^−2^ (Fig. 4b), compared to 0.076 C m^−2^ in P&F with low *M*_w_ (Fig. 4b). Consistently, the P&F samples with high *M*_w_ (670–700 kg mol^−1^) exhibited lower piezoelectric *d*_33_ coefficients (−3.0/+4.2 compared to −10.8/+12.0 of P&F samples with low *M*_w_ of 180 kg mol^−1^) after the ferroelectric measurement up to 300 kV mm^−1^. No obvious piezoelectric resonance peak could be detected in the frequency dependence of the dielectric spectra of the poled 670–700 kg mol^−1^ P&F films, while a small but clear peak (thickness extension mode) can be seen for the poled 180 kg mol^−1^ P&F films (Supplementary Fig. 8). This confirms the existence of readily reversible polar structures in high *M*_w_ P&F films. At high temperatures, the small-sized polar structures in P&F samples suffer severe thermal fluctuation and become even more unstable, as highlighted by the temperature dependence of the dielectric permittivity (Supplementary Fig. 9) and ferroelectric hysteresis loops measured at high temperatures (Supplementary Fig. 10), where the dielectric constant showed a declining trend at temperatures above 50 °C and the disappearance of current peaks (*P*_1_′ and *P*_2_′) upon the removal of electric fields (>140 °C).

## Discussion

In summary, we have demonstrated a facile, readily implementable and scalable process, P&F, to produce PVDF with good ferroelectric properties. Only seven P&F cycles are needed to transform the non-polar α-phase PVDF into the predominantly ferroelectric β-phase PVDF (~98%). The formation of the β-phase is proposed to be a result of a pressure-induced phase transformation, and importantly, more effective stress transfer during P&F. Moreover, studies of the ferroelectric behaviour revealed relaxor-like ferroelectricity in P&F PVDF films with high *M*_w_ (534 and 670–700 kg mol^−1^), which is attributed to reversible field-induced structural change induced by the thermally unstable small-sized polar structures, as suggested by the decreasing crystallite size (down to ~4 nm) after P&F. An ultrahigh energy density of 35 J cm^−3^ was achieved at 880 kV mm^−1^ in a P&F PVDF film with *M*_w_ of 670–700 kg mol^−1^, which is the highest value reported for a polymeric dielectric capacitor, to the best of our knowledge, and well beyond the value for commercial electrochemical capacitors (20–29 J cm^−3^). This finding promises to have a significant impact on the field of pulse power applications and could produce a step change in the field of dielectric capacitors, so far limited by their low energy storage density.

## Methods

### Materials and film preparation

PVDF pellets or powders with different *M*_w_ of 180 and 534 kg mol^−1^ were purchased from Sigma-Aldrich Chemical Co. PVDF with *M*_w_ of 670–700 kg mol^−1^ (Solef^®^ 6020) was purchased from Solvay S.A. The initial HP films with an average area of 5 cm × 5 cm were prepared using a Collin hot-press P300E (Germany) at 180 °C and 113 kN for 2 min followed by cold water quenching to room temperature. The initial films were first folded and cold pressed at 300 kN. The temperature was then increased from ambient temperature to 165 °C. The folded films were then held for 5 min under the conditions of 165 °C and 300 kN, followed by cold water quenching with the pressure maintained. The overall thickness of the P&F films increases with the number of P&F cycles. For instance, the films thickness increases from 250 to 350 μm for HP film to 1.0–1.2 mm for films after seven cycles.

### Microstructure characterization

FTIR spectroscopy (Tensor 27, Bruker Optik GmbH, Ettlingen, Germany) and wide-angle XRD with Cu/Kα radiation (wavelength 0.15418 nm) (X’Pert Pro, PANalytical, Almelo, The Netherlands) were used to characterize the crystalline structure of the samples. Thermal analysis was performed using differential scanning calorimeter (DSC) (DSC 25, TA Instruments, Asse, Belgium) with a heating/cooling rate of 10 °C min^−1^. The degrees of crystallinity of films were determined via measuring the densities of samples based on the Archimedes principle. The densities of amorphous PVDF, pure α- and pure β-PVDF are 1.68, 1.92 and 1.97 g cm^−3^, respectively^39,40^. The morphology of samples was characterized using a scanning electron microscopy (FEI Inspect-F, Hillsboro, OR, USA) and AFM (NT-MDT, Ntegra systems, Russia). The AFM images were obtained using tapping mode and recorded horizontally from left up to right bottom at a scanning rate of 1 Hz with pixel scan of 256×256. Nanoprobe cantilevers (PPP-NCHR, Nanosensors, Neuchatel, Switzerland) designed for non-contact AFM mode were used to acquire images.

### Functional properties characterization

The temperature and frequency dependence of dielectric properties were characterized using a LCR meter (4284A, Agilent, Santa Clara, CA) and Precision Impedance Analyser (4294A, Agilent, Santa Clara, CA), respectively. Unipolar and bipolar ferroelectric hysteresis loops were obtained on a ferroelectric tester (NPL, Teddington, UK) at room temperature using a triangle waveform with a frequency of 10 Hz. The tester can only generate a maximum voltage of 10 kV, which significantly limits the field that can be applied to relatively thick specimens. This is particularly problematic as the thickness of P&F films increases with P&F cycles (1.0–1.2 mm). In order to obtain films of relatively low thickness (~10 μm) comparable to stretched films (~35 μm), a thin layer of PTFE release agent was sprayed on the film surface before each P&F cycle. This allowed the separation of an individual thin layer from the multilayer P&F films. The presence of PTFE did not significantly modify the overall crystalline content, crystalline phase and discharged energy density (Supplementary Fig. 11). The two-parameter Weibull analysis was performed to study the characteristic breakdown strength *E*_b_ of the one single layer of P&F film after seven cycles,

$$P(E) = 1 - {\mathrm{exp}}[ - (E/E_{\mathrm{b}})^{\beta} ].$$

The *E* is the breakdown field of ten samples measured during the ferroelectric hysteresis test, *E*_b_ is characteristic breakdown strength at which the probability of dielectric breakdown is 63.2%, *P*(*E*) is the statistic cumulative probability of dielectric breakdown and *β* is the parameter related to the reliability of films.

### FEM modelling

The commercial package Abaqus Standard 6.14 (Dassault Systemes) was used to conduct simulations for this study. In all simulations, the models were discretized with eight-node brick element with reduced integration (C3D8R). A static analysis with large deformation formulation was performed; the material behavior was captured using a NeoHookean material perfectly plastic after the yield point. Contact between surfaces employed the Coulomb model of friction. Symmetry planes were used for the model geometry.

## Supplementary information

Supplementary Information

## Data Availability

The authors declare that the data supporting the findings of this study are available within the article and its Supplementary Information files and also are available from the corresponding authors upon reasonable request.

## References

[1] Hao X (2013). A review on the dielectric materials for high energy-storage application. J. Adv. Dielectr..

[2] Christen T, Carlen MW (2000). Theory of ragone plots. J. Power Sources.

[3] Kousksou T, Bruel P, Jamil A, El Rhafiki T, Zeraouli Y (2014). Energy storage: applications and challenges. Sol. Energy Mater. Sol. Cells.

[4] Sarjeant WJ, Zirnheld J, MacDougall FW (1998). Capacitors. IEEE Trans. Plasma Sci..

[5] Yao Z (2017). Homogeneous/inhomogeneous-structured dielectrics and their energy-storage performances. Adv. Mater..

[6] Burke A (2007). R&D considerations for the performance and application of electrochemical capacitors. Electrochim. Acta.

[7] Sun H, Fu X, Xie S, Jiang Y, Peng H (2016). Electrochemical capacitors with high output voltages that mimic electric eels. Adv. Mater..

[8] Dang Z-M, Yuan J-K, Yao S-H, Liao R-J (2013). Flexible nanodielectric materials with high permittivity for power energy storage. Adv. Mater..

[9] Horiuchi S, Tokura Y (2008). Organic ferroelectrics. Nat. Mater..

[10] Jiang J (2019). Polymer nanocomposites with interpenetrating gradient structure exhibiting ultrahigh discharge efficiency and energy density. Adv. Energy Mater..

[11] Lovinger AJ (1983). Ferroelectric polymers. Science.

[12] Meng N (2016). Processing and characterization of free standing highly oriented ferroelectric polymer films with remarkably low coercive field and high remnant polarization. Polymer.

[13] Ribeiro C (2018). Electroactive poly(vinylidene fluoride)-based structures for advanced applications. Nat. Protoc..

[14] Katsouras I (2015). The negative piezoelectric effect of the ferroelectric polymer poly(vinylidene fluoride). Nat. Mater..

[15] Meng N, Zhu X, Mao R, Reece MJ, Bilotti E (2017). Nanoscale interfacial electroactivity in PVDF/PVDF-TrFE blended films with enhanced dielectric and ferroelectric properties. J. Mater. Chem. C.

[16] Gomes J, Nunes JS, Sencadas V, Lanceros-Mendez S (2010). Influence of the β-phase content and degree of crystallinity on the piezo- and ferroelectric properties of poly(vinylidene fluoride). Smart Mater. Struct..

[17] Wu J (2018). Perovskite Srx(Bi1−*x*Na0.97−*x*Li0.03)0.5TiO_3_ ceramics with polar nano regions for high power energy storage. Nano Energy.

[18] Zhang QM, Bharti V, Zhao X (1998). Giant electrostriction and relaxor ferroelectric behavior in electron-irradiated poly(vinylidene fluoride-trifluoroethylene) copolymer. Science.

[19] Bobnar V (2003). Dielectric properties of relaxor-like vinylidene fluoride−trifluoroethylene-based electroactive polymers. Macromolecules.

[20] Xu H (2001). Ferroelectric and electromechanical properties of poly(vinylidene-fluoride–trifluoroethylene–chlorotrifluoroethylene) terpolymer. Appl. Phys. Lett..

[21] Guan F (2011). Confined ferroelectric properties in poly(vinylidene fluoride‐co‐chlorotrifluoroethylene)‐graft‐polystyrene graft copolymers for electric energy storage applications. Adv. Funct. Mater..

[22] Li W (2010). Electric energy storage properties of poly(vinylidene fluoride). Appl. Phys. Lett..

[23] Prateek Thakur VK, Gupta RK (2016). Recent progress on ferroelectric polymer-based nanocomposites for high energy density capacitors: synthesis, dielectric properties, and future aspects. Chem. Rev..

[24] Qi L, Petersson L, Liu T (2014). Review of recent activities on dielectric films for capacitor applications. J. Int. Counc. Electr. Eng..

[25] Ren X, Meng N, Yan H, Bilotti E, Reece MJ (2019). Remarkably enhanced polarisability and breakdown strength in PVDF-based interactive polymer blends for advanced energy storage applications. Polymer.

[26] Tan Q, Irwin P, Cao Y (2006). Advanced dielectrics for capacitors. IEEJ Trans. Fund. Mater..

[27] Wang Y, Zhou X, Chen Q, Chu B, Zhang Q (2010). Recent development of high energy density polymers for dielectric capacitors. IEEE Trans. Dielectr. Electr. Insulation.

[28] Wu S (2013). Aromatic polythiourea dielectrics with ultrahigh breakdown field strength, low dielectric loss, and high electric energy density. Adv. Mater..

[29] Zhang X (2016). Achieving high energy density in PVDF-based polymer blends: suppression of early polarization saturation and enhancement of breakdown strength. ACS Appl. Mater. Interfaces.

[30] Zhang Z, Chung TCM (2007). Study of VDF/TrFE/CTFE terpolymers for high pulsed capacitor with high energy density and low energy loss. Macromolecules.

[31] Zhou X (2009). Electrical breakdown and ultrahigh electrical energy density in poly(vinylidene fluoride-hexafluoropropylene) copolymer. Appl. Phys. Lett..

[32] Santagiuliana G (2018). Breaking the nanoparticle loading–dispersion dichotomy in polymer nanocomposites with the art of croissant-making. ACS Nano.

[33] Martins P, Lopes AC, Lanceros-Mendez S (2014). Electroactive phases of poly(vinylidene fluoride): determination, processing and applications. Prog. Polym. Sci..

[34] Hu ZJ, Tian MW, Nysten B, Jonas AM (2009). Regular arrays of highly ordered ferroelectric polymer nanostructures for non-volatile low-voltage memories. Nat. Mater..

[35] Meng N (2017). Crystallization kinetics and enhanced dielectric properties of free standing lead-free PVDF based composite films. Polymer.

[36] Cai X, Lei T, Sun D, Lin L (2017). A critical analysis of the α, β and γ phases in poly(vinylidene fluoride) using FTIR. RSC Adv..

[37] Benz M, Euler WB (2003). Determination of the crystalline phases of poly(vinylidene fluoride) under different preparation conditions using differential scanning calorimetry and infrared spectroscopy. J. Appl. Polym. Sci..

[38] Li Y (2014). Multiple stage crystallization of gamma phase poly(vinylidene fluoride) induced by ion–dipole interaction as revealed by time-resolved FTIR and two-dimensional correlation analysis. Polymer.

[39] Ameduri B (2009). From vinylidene fluoride (VDF) to the applications of VDF-containing polymers and copolymers: recent developments and future trends. Chem. Rev..

[40] Hasegawa R, Takahashi Y, Chatani Y, Tadokoro H (1972). Crystal Structures of three crystalline forms of poly(vinylidene fluoride). Polym. J..

[41] Li M (2013). Revisiting the δ-phase of poly(vinylidene fluoride) for solution-processed ferroelectric thin films. Nat. Mater..

[42] Hasegawa R, Kobayashi M, Tadokoro H (1972). Molecular conformation and packing of poly(viny1idene fluoride). Stability of three crystalline forms and the effect of high pressure. Polym. J..

[43] Siesler HW (1985). Rheo-optical Fourier-transform infrared spectroscopy of polymers. 9. Stretching-induced II(α)-I(β) crystal phase transformation in poly(vinylidene fluoride). J. Polym. Sci..

[44] Sencadas V, Gregorio R, Lanceros-Méndez S (2009). α to β Phase transformation and microestructural changes of PVDF films induced by uniaxial stretch. J. Macromol. Sci. Part B.

[45] Martín J (2017). Solid-state-processing of δ-PVDF. Mater. Horiz..

[46] Sharma M, Madras G, Bose S (2014). Process induced electroactive β-polymorph in PVDF: effect on dielectric and ferroelectric properties. PCCP.

[47] Chow TS (1992). Stress–strain behavior of polymers in tension, compression, and shear. J. Rheol..

[48] Walley SM, Field JE (1994). Strain rate sensitivity of polymers in compression from low to high rates. DYMAT J..

[49] Kang SJ (2007). Localized pressure-induced ferroelectric pattern arrays of semicrystalline poly(vinylidene fluoride) by microimprinting. Adv. Mater..

[50] Martín J (2017). Relaxations and relaxor-ferroelectric-like response of nanotubularly confined poly(vinylidene fluoride). Chem. Mater..

[51] Furukawa T, Date M, Fukada E (1980). Hysteresis phenomena in polyvinylidene fluoride under high electric field. J. Appl. Phys..

[52] Yang L (2014). Relaxor ferroelectric behavior from strong physical pinning in a poly(vinylidene fluoride-co-trifluoroethylene-co-chlorotrifluoroethylene) random terpolymer. Macromolecules.

[53] Tan X, Ma C, Frederick J, Beckman S, Webber KG (2011). The antiferroelectric ↔ ferroelectric phase transition in lead-containing and lead-free perovskite ceramics. J. Am. Ceram. Soc..

[54] Kan D, Anbusathaiah V, Takeuchi I (2011). Chemical substitution-induced ferroelectric polarization rotation in BiFeO_3_. Adv. Mater..

[55] Oka Y, Koizumi N, Murata Y (1986). Ferroelectric order and phase transition in polytrifluoroethylene. J. Polym. Sci. Part B.

[56] Furukawa T, Takahashi Y (2001). Ferroelectric and antiferroelectric transitions in random copolymers of vinylidene fluoride and trifluoroethylene. Ferroelectrics.

[57] Woo J, Hong S, Min DK, Shin H, No K (2002). Effect of domain structure on thermal stability of nanoscale ferroelectric domains. Appl. Phys. Lett..

[58] Furukawa T (1989). Ferroelectric properties of vinylidene fluoride copolymers. Phase Transit..

