# Supplementary material for: Ultrahigh β-phase content poly(vinylidene fluoride) with relaxor-like ferroelectricity for high energy density capacitors
Source: Nat Commun. 2019 Oct 18;10:4535. doi: 10.1038/s41467-019-12391-3 (PMC6800420; doi:10.1038/s41467-019-12391-3)
Supplement: Supplementary file 1 — Supplementary Information [file 41467_2019_12391_MOESM1_ESM.pdf]

Supplementary Information

for

Ultrahigh β-phase content poly(vinylidene fluoride) with relaxor-like ferroelectricity for high energy density capacitors

Authors: Nan Meng[1], Xintong Ren[1], Giovanni Santagiuliana[1], Leonardo Ventura[1], Han Zhang[1,2], Jiyue Wu[1], Haixue Yan[1,2], Michael J Reece[1,2], Emiliano Bilotti[1,2*]

1. School of Engineering and Materials Science, Queen Mary University of London, Mile End Road, E1 4NS, UK

2. NPU-QMUL Joint Research Institute of Advanced Materials and Structures, Queen Mary University of London, Mile End Road, London E1 4NS

[*]E-mail: e.bilotti@qmul.ac.uk

Supplementary Notes

1. The phase deterimination of PVDF using FTIR is summarized as follows:

(1) Check characteristic peaks (especially exclusive peaks to a particular phase) (Table S2):

α-phase: 764 cm^{-1}, 975 cm^{-1} and 1212 cm^{-1}; β-phase: 840 cm^{-1} and 1275 cm^{-1} (1275 cm^{-1} is exclusive to β-phase); γ-phase: 812, 832, 840 and 1234 cm^{-1} (1234 cm^{-1} is exclusive to γ-phase).

(2) Calculation:

(i) For a sample containing only α- and β-phase. The fraction of β-phase ($F(\beta)$) can be calculated using the following equations: $F(\beta)=\frac{A_\beta}{\left(\frac{K_\beta}{K_\alpha}\right)A_\alpha+A_\beta}\times 100\%=\frac{A_\beta}{1.26\times A_\alpha+A_\beta}\times 100\%$, where A_α and A_β are the absorbance at 764 and 840 cm^{-1}, respectively. K_α and K_β are the corresponding absorbance coefficients, 6.1×10^4 and 7.7×10^4 $cm^2\ mol^{-1}$, respectively. The fraction of α-phase ($F(\alpha)$): $F(\alpha)=1-F(\beta)$.

(ii) For a sample containing only α- and γ-phase. The fraction of γ-phase ($F(\gamma)$) can be calculated using the following equations: $F(\gamma)=\frac{A_\gamma}{\left(\frac{K_\gamma}{K_\alpha}\right)A_\alpha+A_\gamma}\times 100\%=\frac{A_\gamma}{0.41\times A_\alpha+A_\gamma}\times 100\%$, where A_α and A_γ are the absorbance at 764 and 832 cm^{-1}, respectively. K_α and K_γ are the corresponding absorbance coefficients, 0.365 and 0.150 μm^{-1}, respectively. $F(\alpha)$ is calculated using $1-F(\gamma)$.

(iii) For a sample containing α-, β- and γ –phase, firstly calculate the content of the polar phases (F(β,γ)) using: $F(\beta,\gamma)=\frac{A_{\beta,\gamma}}{\left(\frac{K_{\beta,\gamma}}{K_{\alpha}}\right)A_{\alpha}+A_{\beta,\gamma}}=\frac{A_{\beta,\gamma}}{1.26\times A_{\alpha}+A_{\beta,\gamma}}$, where A_{α} and $A_{\beta,\gamma}$ are the absorbance at 764 and 840 cm^{-1}, respectively. K_{α} and $K_{\beta,\gamma}$ are the corresponding absorbance coefficients, 6.1 × 10^4 and 7.7 × 10^4 cm^2 mol^{-1}, respectively. F(α) is calculated using 1− F(β,γ). F(β) and F(γ) are calculated using the absorbance of peaks at 1275 cm^{-1} (β-phase) and 1234 cm^{-1}(γ-phase): $F(\beta)=F(\beta,\gamma)\times\frac{A_{1275}}{A_{1275}+A_{1234}}\times 100\%$. $F(\gamma)=F(\beta,\gamma)\times\frac{A_{1234}}{A_{1275}+A_{1234}}\times 100\%$

2. Modelling and investigation of the mechanism of phase transformation

The sample thickness increases with the number of P&F cycles. However, at the end of each pressing step it decreases by a factor h: $H_f = h\times H_i$, where $H_i(n)=\frac{(2h)^n}{h}H_0$ is the sample thickness after folding but before pressing, H_0 is the thickness of initial HP sample before P&F, n the number of P&F cycles, and h = 0.61 ± 0.09. Similarly, the sample area decreases with the number of cycles, but it increases by a factor a = 1.65 ± 0.14 after each pressing compared to its value just before each pressing: $A_f(n)=a\times A_i(n)=\left(\frac{a}{2}\right)^n A_0$, where A_0 is the initial sample area. This happens because the sample is deformed plastically at each cycle, but its volume remains constant. As a consequence of the dimensional changes, the nominal stress applied to the sample at the end of each cycle is $P_i(n)=P_1\left(\frac{2}{a}\right)^{n-1}$, where $P_1=\frac{F}{A_0/2}$, while the true stress at the end of each cycle is $P_f(n)=\frac{P_i(n)}{a}$. Approximately (assuming a plastic deformation with a constant stress), the energy adsorbed to plastically deform the sample volume at each n cycle is $\omega(n)=\frac{E(n)}{V}\approx\frac{F\Delta H(n)}{A_0H_0}=\frac{F}{A_0}\frac{1-h}{h}(2h)^n=P_1(1-h)(2h)^{n-1}$. Therefore, the total amount of adsorbed energy (considering the energy adsorbed in the previous cycles) is $\Omega(n)=\sum_{x=1}^{n}\omega(x)=P_1(1-h)\frac{1-(2h)^n}{1-2h}$. The factors that can influence the amount of Ω adsorbed per cycle, or the proportionality between f_β and Ω (or n) are:

- Strain-rate. Higher strain-rates allow for higher Ω. However, the closing speed of our hot-press cannot be changed and is constant (v_{HP} = 1.8 mm s^{-1}), but the sample thickness increases with the folding, thus the nominal strain-rate should decrease during the process: $\dot{\varepsilon}(n) = h\frac{v_{HP}}{H_0}(2h)^{-n}$, i.e. the increment of β-phase should be lower and lower with the P&F cycles than what we would expect from Eq. 2 reported in the main paper. However, this range of strain-rates does not seem to be big enough to justify a sensitive deviation of $\Omega(n)$ from the expression presented above: the measured plastic deformation in Figure 2a in the main paper shows a repetitive trend with the cycles, i.e. the coefficients h and a are always the same for any cycle.

- Initial sample area A_0. As $\Omega(n) = P_1(1-h)\frac{1-(2h)^n}{1-2h}$ and $P_1 = \frac{F}{A_0/2}$, smaller values of A_0 will lead to higher plastic energies. We tested samples with three different areas: (50 mm×50 mm), (30 mm×15 mm) and (10 mm×10 mm), which corresponded to applied pressures of 120 MPa, 667 MPa and 3000 MPa. We found that films with smaller values of A_0 presented higher β–phase contents (Figure 3a in the main paper). However, this route is clearly not viable for the production of large-area β–phase PVDF films, so several cycles will always be required in real applications. Note that the effect of using higher compression forces (F) is the same as having a smaller A_0, so we do not consider it.

- Molecular weight (M_w) and annealing temperature (T_{anneal}). Higher M_w represents a higher chain entanglement, thus lower chain mobility. Lower T_{anneal} also induces lower chain mobility. To study their effects, we varied the annealing temperature relative to T_m. Two additional PVDF polymers were studied with different M_w (180 kg mol^{-1} and 534 kg mol^{-1}) but similar T_m (169 °C and 172 °C, respectively) (Figure 3d in the main text), to the PVDF used so far (M_w 670-700 kg mol^{-1}, T_m 172 °C). All of the PVDFs can transform to β-phase with high content (>90%) when annealing below T_m, regardless of M_w (Figure 3e and Supplementary Figure 5). The γ-phase, with mixed components of α- and β-phase, formed

in PVDF with M_w of 180 kg mol^{-1} P&F at 175 °C (T_m+6 °C) and 534 kg mol^{-1} P&F at 180 °C (T_m+8 °C) (Figure 3d), evidenced by the exclusive characteristic FTIR band at 1234 cm^{-1}. The β-phase increased more remarkably during the P&F process for the PVDFs with higher M_w (Figure 3d). PVDF with M_w of 670-700 kg mol^{-1} still showed ultrahigh content of β-phase (>95%) carrying out the P&F at 185 °C (~T_m+15 °C) (Supplementary Figure 5), and no traces of γ-phase was produced even at a temperature of 195 °C (~T_m+20 °C). The phase evolution of PVDF with T_{anneal} and M_w during P&F is explained in Figure 3e. Firstly, P&F can better induce the formation of β-phase in the case of high M_w PVDF (>95% in the 534 and 670-700 kg mol^{-1} P&F samples, while only ~90% in the 180 kg mol^{-1} P&F samples). Apart from the M_w, the role of T_{anneal} can be summarised as follow: a relatively low T_{anneal} (<T_m and as low as room temperature, as shown in Supplementary Figure 5) favours the transition to β-phase; a moderately high T_{anneal} results in the incomplete transition from α- to β- and the formation of γ-phase in low M_w PVDF; an even higher T_{anneal} (>>T_m) makes PVDF maintain the non-polar α-phase. It should be mentioned that films P&F at temperatures below 80 °C showed obvious cracks especially for PVDF with high M_w, therefore, relatively higher annealing temperatures (100-165 °C) are preferred to prepare continuous and high quality P&F films.

Supplementary Figures

Supplementary Figure 1.

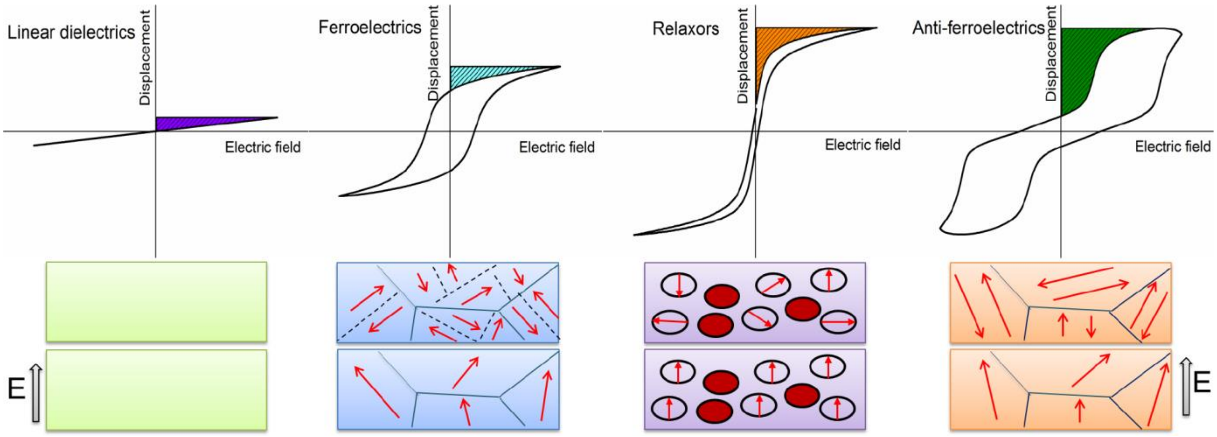

Supplementary Figure 1. The characteristics of energy storage and schematic diagrams of dipoles and domain structure before and after poling for linear dielectrics, ferroelectrics, relaxors and anti-ferroelectrics (Reproduced with permission.[17] 2018, Elsevier). Linear dielectrics do not have domains. Normal ferroelectrics have large domains and exhibit ferroelectric switching, which causes high remnant polarization and energy loss, making them not suitable for energy storage. Relaxors have nanodomains and/or polar nanoregions (concept from ceramic field), and the dipoles are highly mobile and reversible, generating high saturated polarization and low remnant polarization, making them suitable for energy storage. Anti-ferroelectrics have reversible phase transition between the anti-ferroelectric and ferroelectric phases, making them also favourable for electric energy storage applications, as highlighted by the larger area representing the discharged energy density.

Supplementary Figure 2.

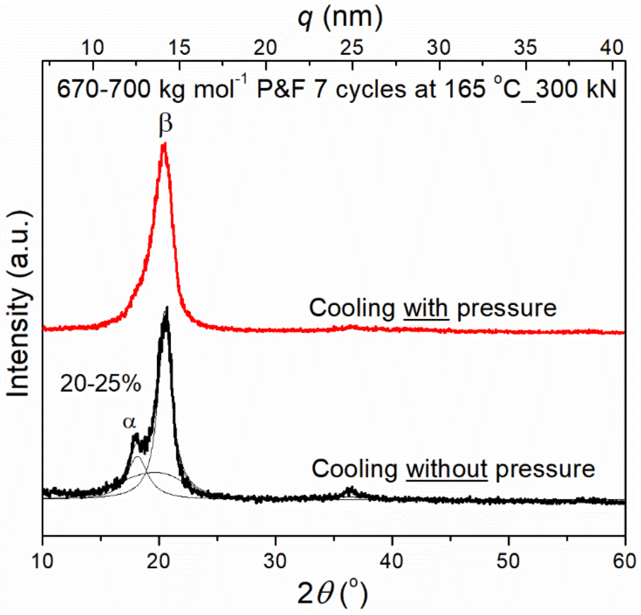

Supplementary Figure 2. XRD data of P&F films after 7 cycles at 165 °C and 300 kN for 5 minutes followed by cold water quenching with and without pressure. The P&F films cooled without pressure show a residual amount of α-phase (20-25 wt. %).

Supplementary Figure 3.

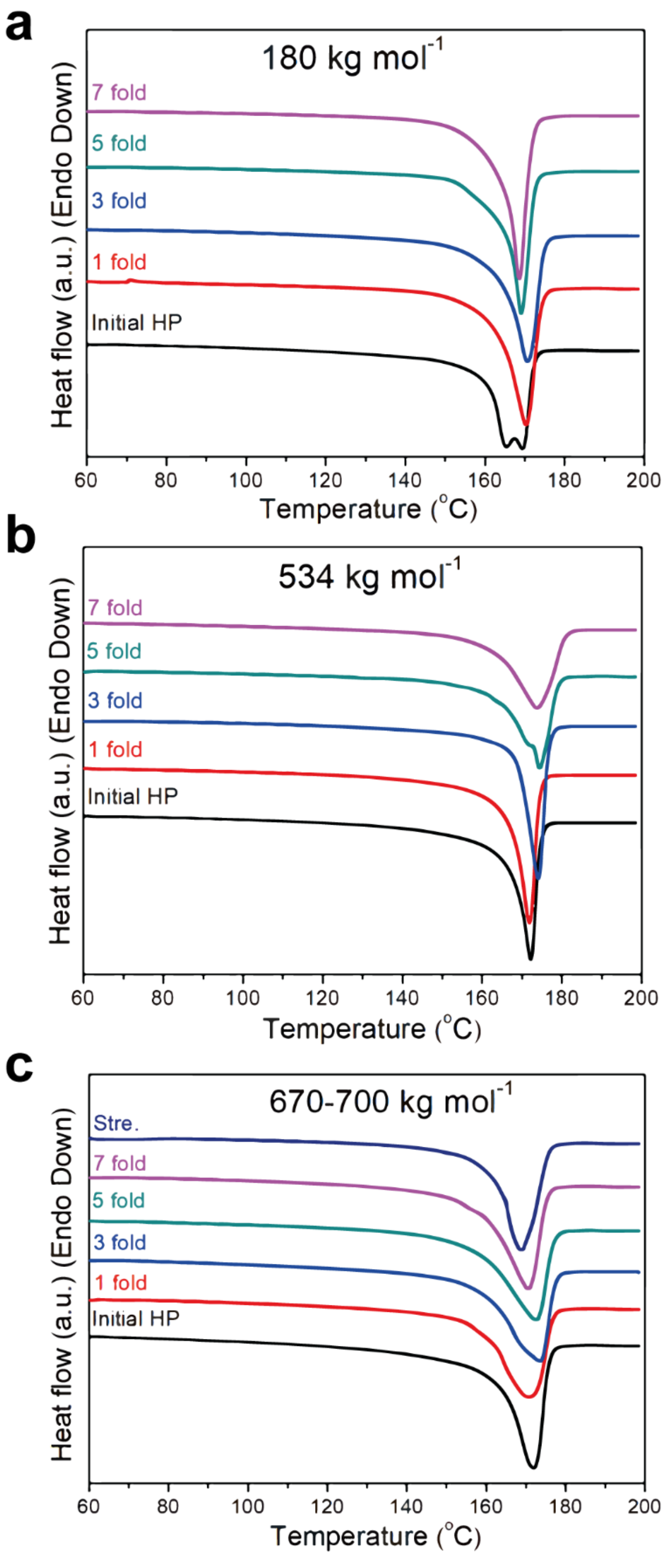

Supplementary Figure 3. DSC curves recorded during the first heating run. (a) M_w of 180 kg mol^{-1}; (b) M_w of 534 kg mol^{-1}and (c) M_w of 670-700 kg mol^{-1}. All of P&F films were produced at 165 °C and 300 kN for 5 minutes.

Supplementary Figure 4.

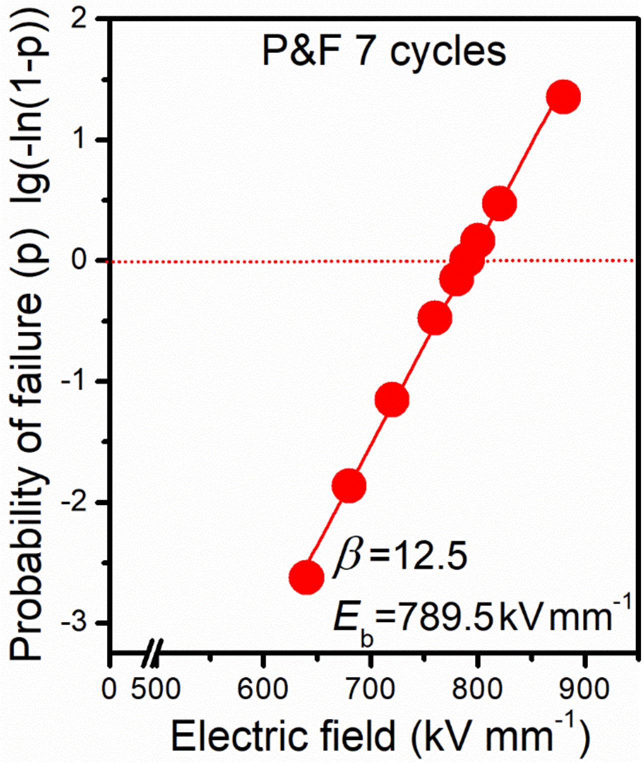

Supplementary Figure 4. The Weibull analysis result of P&F PVDF after seven cycles at 165 °C with M_w of 670-700 kg mol^{-1}. The E_b is 789.5 kV mm^{-1} and the β is 12.5, which indicates that the P&F film is reliable with the applied field up to 789.5 kV mm^{-1}.

Supplementary Figure 5.

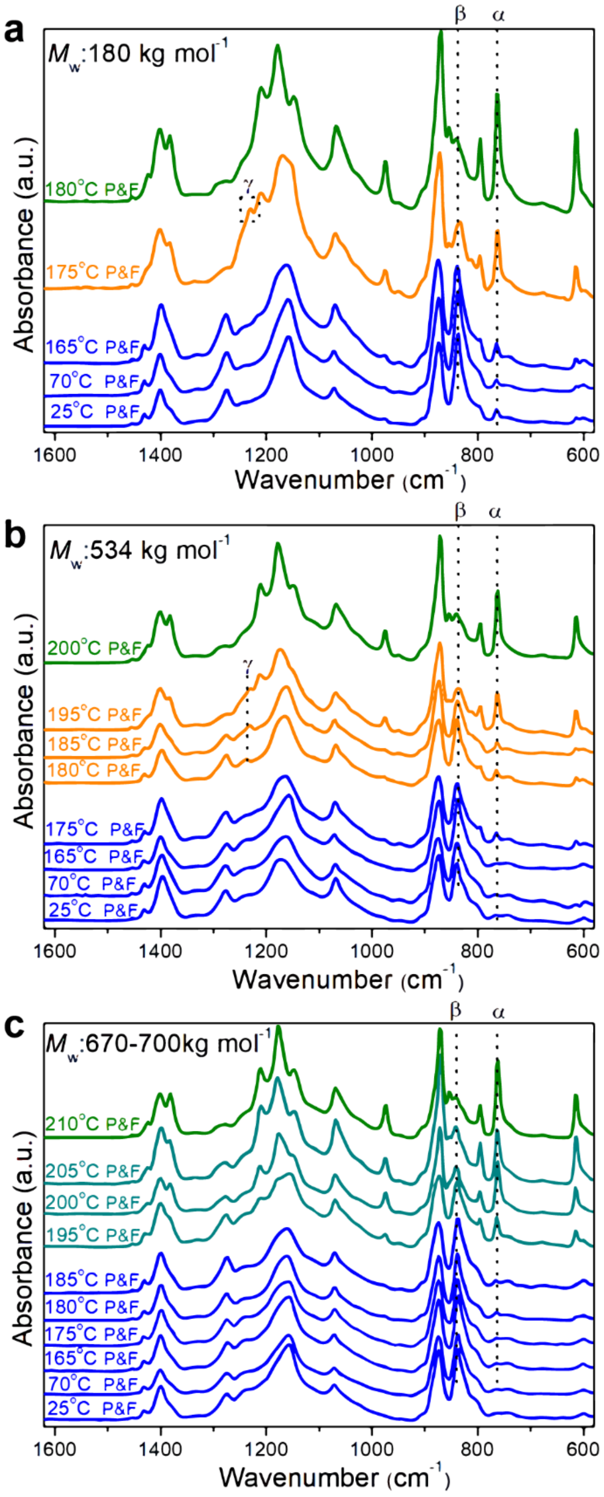

Supplementary Figure 5. FTIR of P&F PVDF samples after seven cycles but at different annealing temperatures (T_{anneal}) and 300 kN for 5 minutes: (a) M_w of 180 kg mol^{-1}; (b) M_w of 534 kg mol^{-1}; and (c) M_w of 670-700 kg mol^{-1}. The transformation to β-phase shows the dependence of T_{anneal}: low T_{anneal} favours the transition to β-phase (blue lines, indicated by the increase of peak intensity at 840 cm^{-1} and decrease of peak intensity at 764 cm^{-1}), moderately high T_{anneal} leads to the formation of γ-phase in PVDF with relatively low M_w (orange lines, 180 and 534 kg mol^{-1}) and incomplete transition in high M_w (cyan lines, 670-700 kg mol^{-1}) and even higher T_{anneal} cannot trigger the phase transition (green lines).

Supplementary Figure 6.

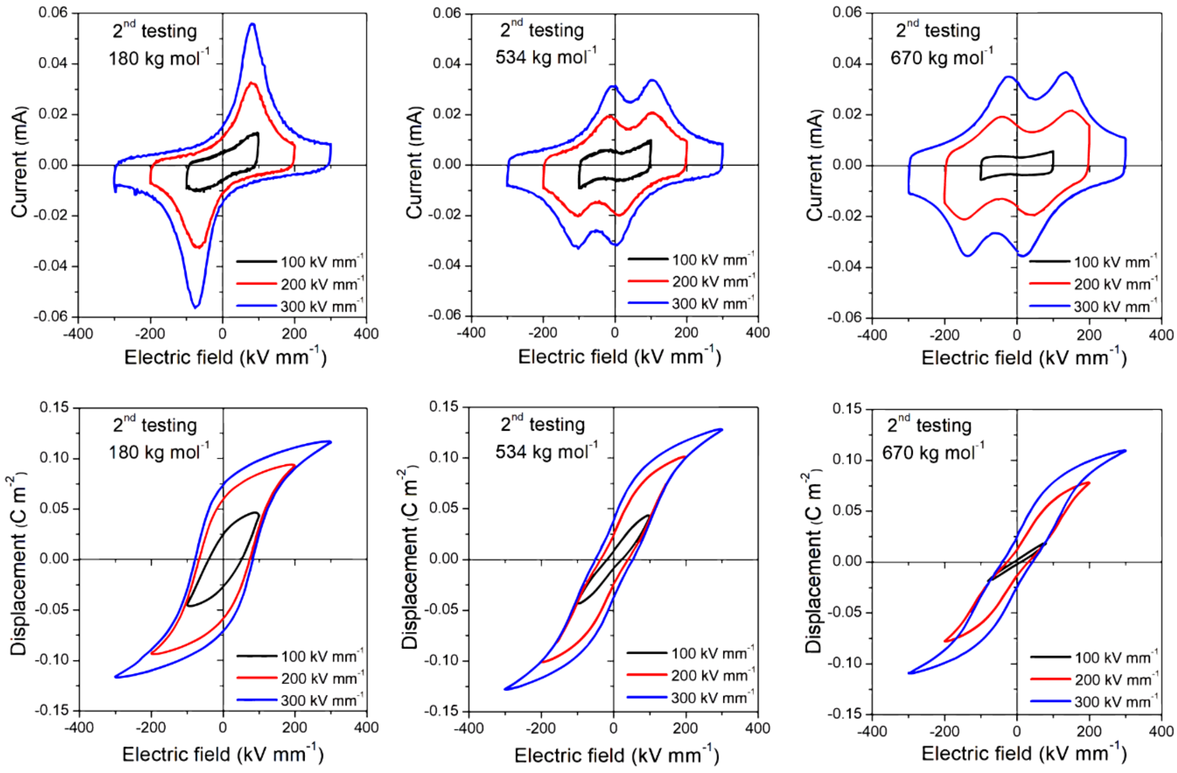

Supplementary Figure 6. Re-measured bipolar ferroelectric Current-Electric field (*I*-*E*) and Displacement-Electric field (*P*-*E*) loops of P&F PVDF with different M_w of 180 kg mol^{-1}, 534 kg mol^{-1} and 670 kg mol^{-1} during the second cycle. Note the different *I*-*E* curve at 200 kV mm^{-1} in P&F samples with M_w of 180 kg mol^{-1}, which suggests that there were field-induced polar structural changes during electric measurement, while there is no obvious change between the first and second measured *I*-*P*-*E* loops in P&F with M_w of 534 and 670 kg mol^{-1}.

Supplementary Figure 7.

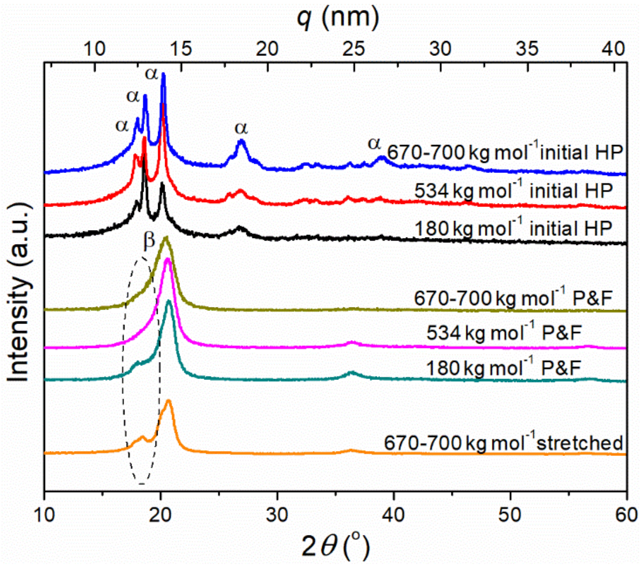

Supplementary Figure 7. XRD data of initial HP films represented by 2θ and q, where $q=(4\pi\sin\theta)/\lambda$ with θ being the half scattering angle, λ being the wavelength of X-ray (0.15418 nm). P&F films after 7 cycles at 165 ℃ and 300 kN for 5 minutes and films solid-state drawn to failure at 100 °C and 10 mm min^{-1}. Combined with FTIR data (Figure 1c), the initial HP films are mainly α-phase, of which the characteristic peaks are at 2θ of about 17.8° $(100)_\alpha$, 18.7° $(020)_\alpha$, 20.2° $(110)_\alpha$, 26.7° $(021)_\alpha$ and 38.9° $(210)_\alpha$ (indexed in figure). P&F samples with low M_w (180 kg mol^{-1}) show a shoulder peak at 2θ=18.0° due to the reflection of $(110)_\alpha$ and $(020)_\alpha$ (highlighted in an ellipse region) coming from the residual non-polar α-phase (~ 10 wt. %). Conversely, the folded samples with high M_w (670 and 534 kg mol^{-1}) only display reflections from the β-phase, $(110)/(200)_\beta$ and $(020)/(101)_\beta$, at 2θ of about 20.7° and 36.5°, respectively. The stretched film, for which the FTIR and ferroelectric *I-P-E* loops are presented in Figure 1d, contains a mixture of α- and β-phases and displays mixed reflections from α- and β-crystals.

Supplementary Figure 8.

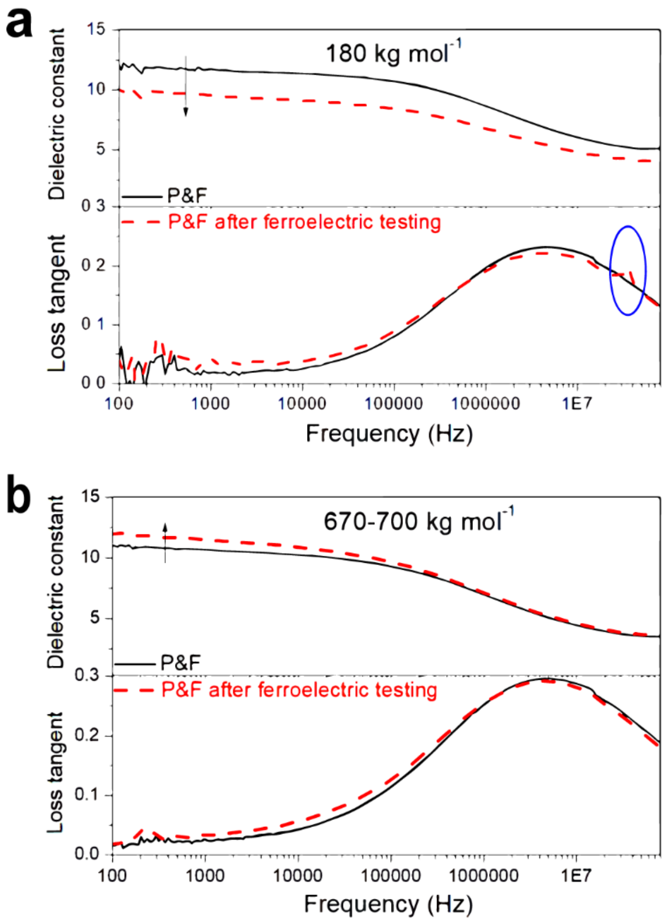

Supplementary Figure 8. The frequency dependence of the dielectric data before and after ferroelectric testing for P&F samples prepared at 165 ℃ and 300 kN for 5 minutes with M_w of: (a) 180 kg mol^{-1}; and (b) 670-700 kg mol^{-1}. The decrease of dielectric constant of P&F films with a M_w of 180 kg mol^{-1} after poling suggests the growth of polar structures. A small but clear piezoelectric resonance peak (thickness extension mode) can be seen in P&F films with a M_w of 180 kg mol^{-1} (highlighted in blue circle). However, P&F films with M_w of 670-700 kg mol^{-1} do not show a piezoelectric resonance peak, which demonstrates the reversibility of the orientation of their dipoles.

Supplementary Figure 9.

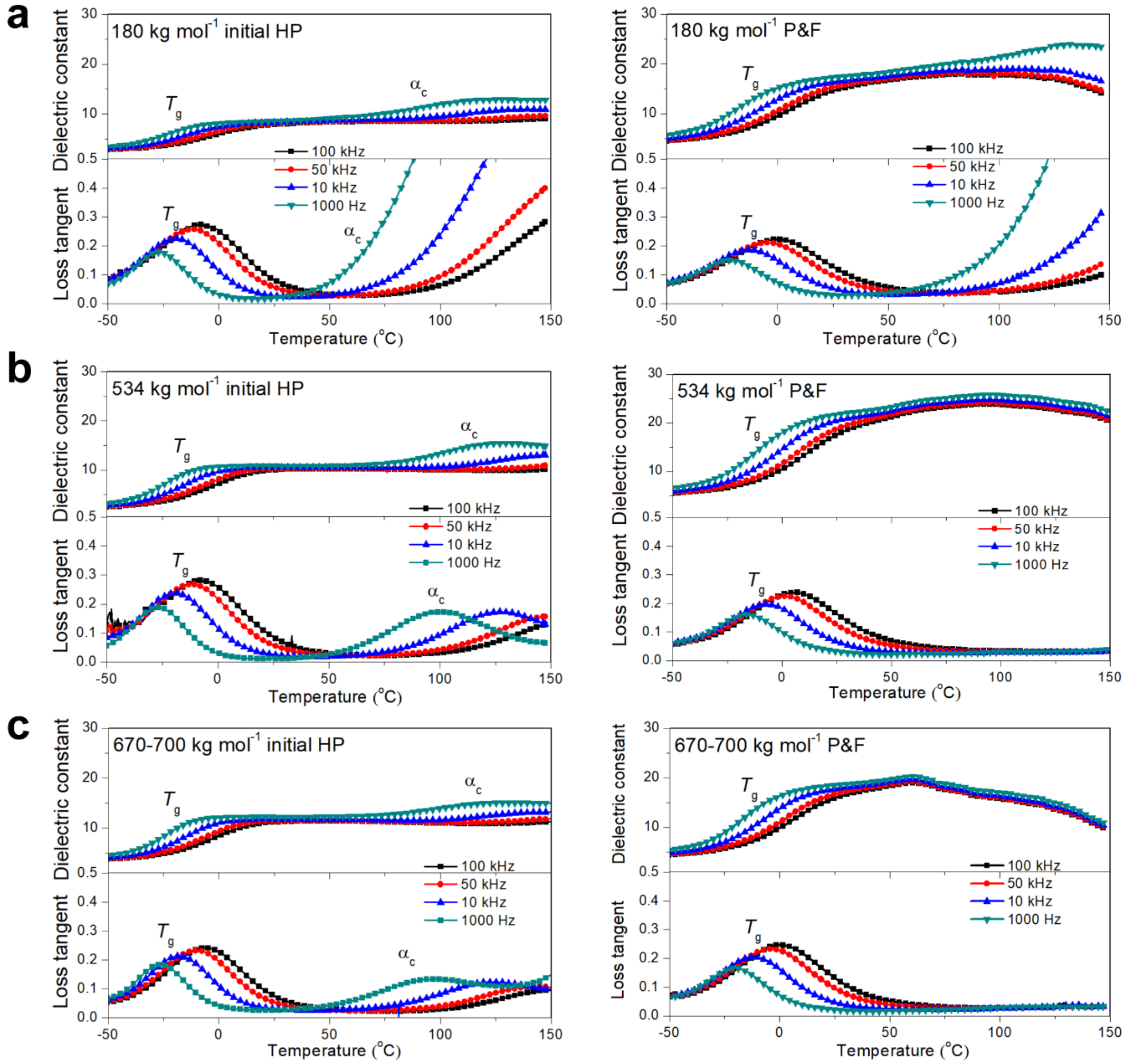

Supplementary Figure 9. The temperature dependence of the dielectric properties of initial HP and P&F PVDF films prepared at 165 ℃ and 300 kN for 5 minutes with different molecular weights**:** (a) 180; (b) 534 and (c) 670-700 kg mol^{-1}. The HP films show two main relaxation behaviours, the low temperature glass transition (~-8 °C at 100 kHz) and high temperature α_c relaxation (~50-150 °C) originating from the motions of polymer chains in α-crystals. The glass transition temperature (T_g) hardly varied (~-25 °C at 1000 Hz) for the films with different M_w. However, the α_c relaxation loss peak in PVDF with M_w of 180 kg mol^{-1} is not evident because it is merged with the abrupt increase of loss tangent at temperatures higher than 50 °C, demonstrating the high mobility of polymer chains in PVDF with low M_w. The P&F films show T_g but no α_c relaxation, which highlights the phase transformation from α- to β-phase. Moreover, P&F film with highest M_w (670-700 kg mol^{-1}) exhibited a broad maximum of dielectric constant at ~ 60 °C, which resembles the behaviour of relaxor ferroelectrics

Supplementary Figure 10.

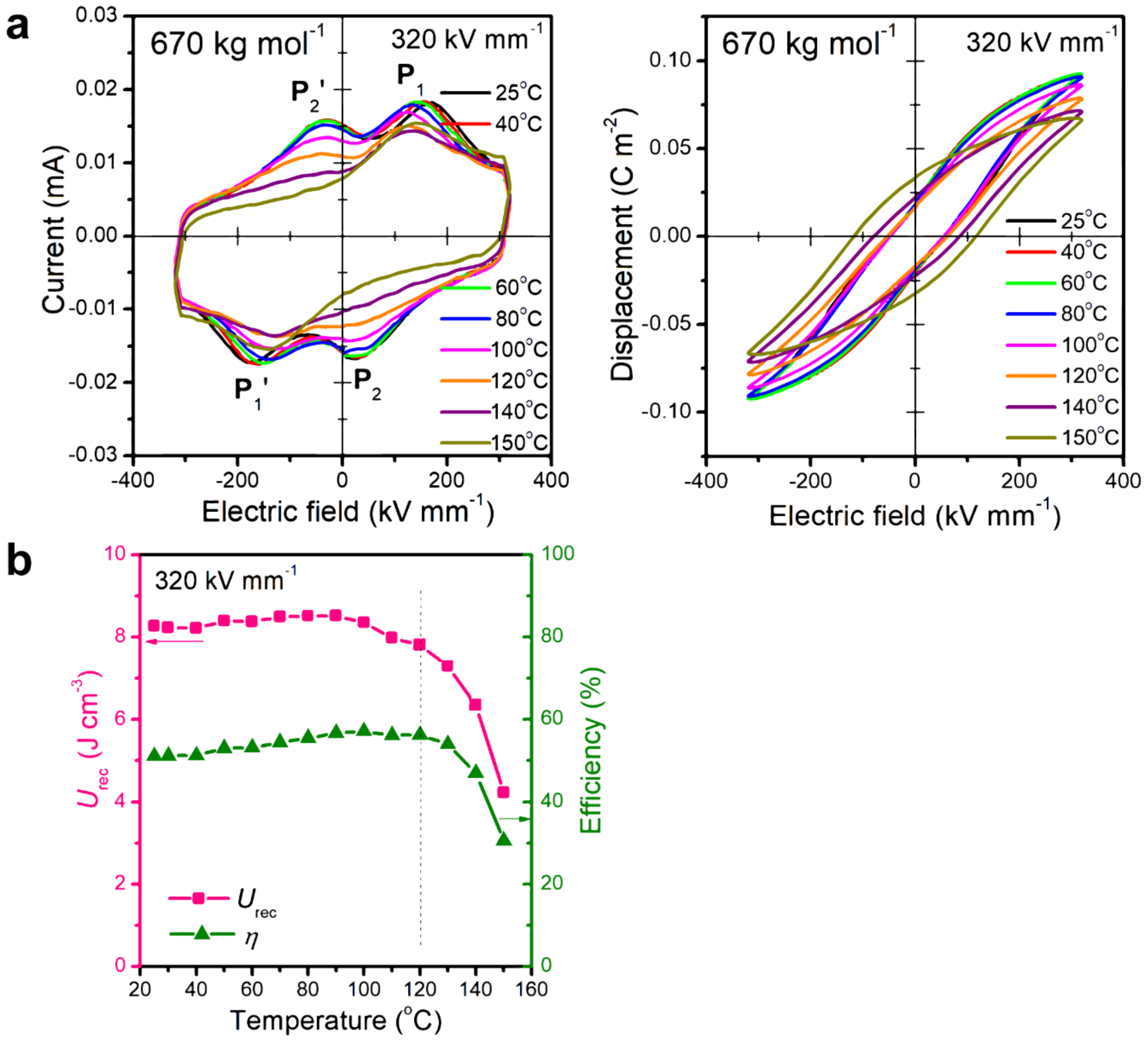

Supplementary Figure 10. Ferroelectric behaviours and electric energy storage properties at high temperatures. (a) Bipolar ferroelectric hysteresis loops measured at 320 kV/mm in the temperature range of 25-150 °C; (b) electric energy storage density and efficiency of P&F PVDF measured at 320 kV/mm in the temperature range of 25-150 °C. With increasing testing temperature, the P_1 peak shifted to lower electric fields demonstrating easier ferroelectric switching and the P_2 peaks became less obvious, demonstrating that the materials changed at high temperatures. Judging from the electric energy storage density and efficiency, P&F PVDF films can be stable at temperatures of about 120 °C, which is reasonable, being higher compared to the mentioned state-of-art commercial BOPP (80-105 °C).

Supplementary Figure 11.

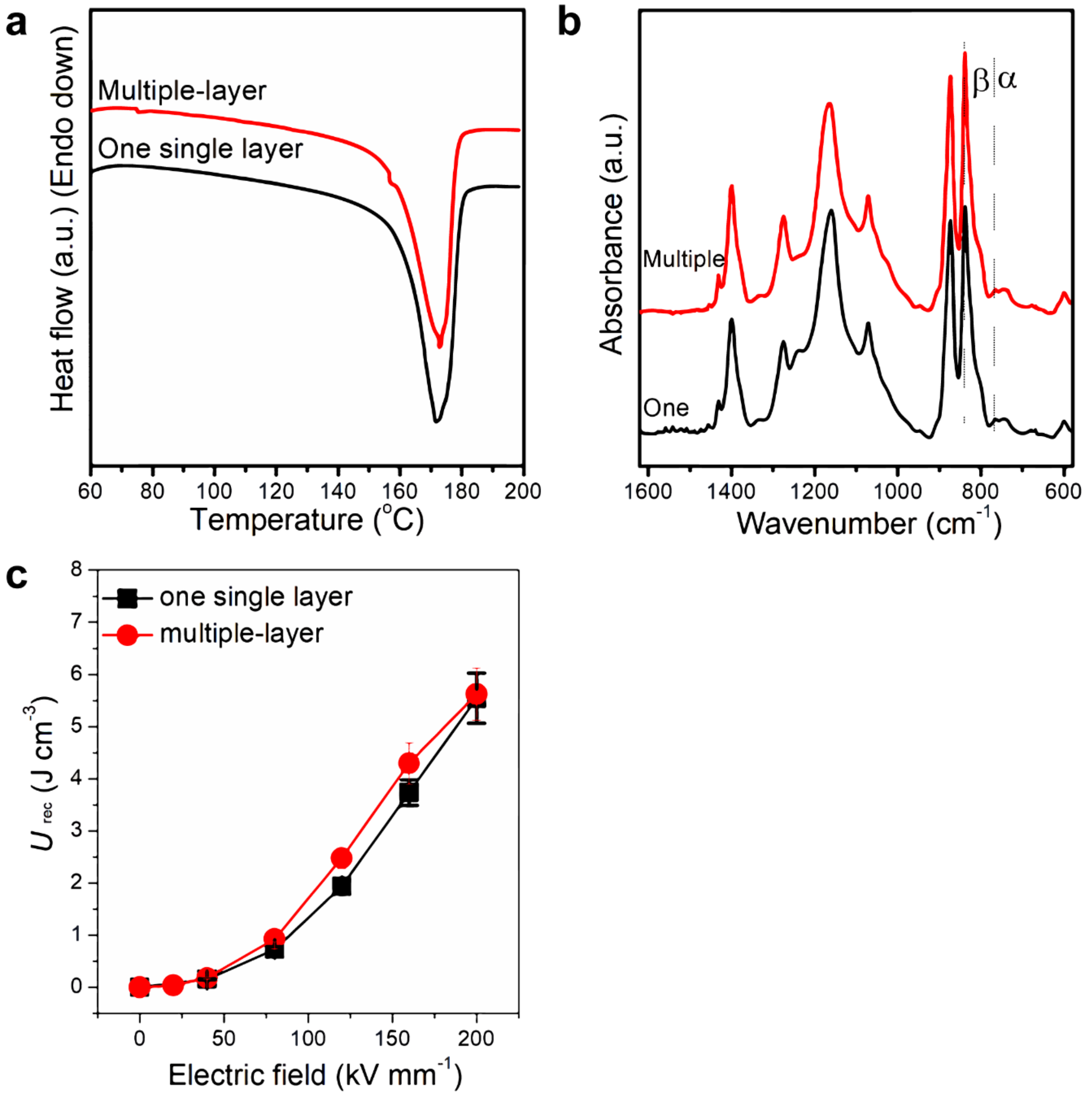

Supplementary Figure 11. The comparison of: (a) DSC curves of first heating cycle; (b) FTIR spectra; (c) discharged energy storage density U_{rec} up to an electric field of 200 kV mm^{-1} for a single layer film peeled off from seven fold PVDF P&F at 165 °C with PTFE release agent and multiple-layer (four) films peeled off from a seven fold PVDF P&F film prepared at 165 °C without PTFE with M_w of 670-700 kg mol^{-1}. Both single layer and multiple-layer films exhibit similar crystallinity of 37±2% and fraction of β phase ~98%. The U_{rec} of both samples is ~5.5 J/cm^3 at 200 kV mm^{-1}. These results demonstrate the existence of PTFE does not influence the structure or ferroelectric properties of P&F films.

Supplementary Tables

Supplementary Table 1. Dielectric properties of various polymers

	Materials	ε' (1 kHz)	tanδ (1 kHz)	E_b (kV/mm)	U_{rec} (J/cm^3)	Maximum Working Temperature (°C)	Reference
Non-polar	Low-density polyethylene (LDPE)	2.3	0.003	350-600	NA	90	[23]
	High-density polyethylene (HDPE)	2.3	0.0005	650-700	NA	90	[23, 59]
	Polytetrafluoroethylene (PTFE)	2	0.0001	88-296	NA	260	[23, 26]
Polar polymers (linear dielectrics)	Polypropylene (PP)	2.2	0.0002	600-750	1-3	80-105	[24, 26]
	Poly(ethylene terephthalate) (PET)	3.3	0.005	570	1-1.5	125	[24]
	Polyphenylene sulfide (PPS)	3	0.0005	560	1-1.5	150	[24, 26]
	Polycarbonate (PC)	2.7-3.2	0.0015	530	0.5-1.0	125	[24, 26]
	Polyimide-Kapton®	3.4	0.002	300	0.5-1.0	260	[23, 26]
	Aromatic polythiourea (ArPTU)	4.2	0.005	1000	22	150	[28]
Polar polymers (PVDF based ferroelectrics)	PVDF	8-12	0.03-0.05	400-600	5-14	100-120	[22, 24, 26]
	PVDF-HFP	15	0.04	700	25	100	[25, 31]
	PVDF-CTFE	13	0.03	620	25	100-120	[27, 60]
	PVDF-TrFE-CFE	52	0.1	400	10	50-60	[61, 62]
	PVDF-TrFE-CTFE	50-60	0.08	500	13	50-60	[25, 30]
	PVDF-TrFE-CFE/PVDF 60/40 blends	36	0.06	640	19.6	50-60	[29]
	PVDF-TrFE-CTFE/PVDF-HFP 50/50 blends	25	0.05	600	21.9	50-60	[25]
	**P&F PVDF**	**11**	**0.02**	**880**	**35**	**120**	**Current work**

ε': dielectric constant; tan*δ*: dielectric loss tangent; E_b: breakdown field; U_{rec}: electric energy storage density.

Supplementary Table 2. FTIR characteristic peaks of PVDF

Presented peaks at (cm^{-1})	No Peaks at (cm^{-1})	Phase
764, 975 and 1212	840	α-phase
840 and 1275	764, 975, 1212 and 1234	β-phase
812, 832 and 1234	764, 975, 1212 and 1275	γ-phase
764, 975, 1212 & 840, 1275	1234	α- and β-phase
764, 975, 1212 & 812, 832, 1234	1275	α- and γ -phase
764, 975, 1212 & 840, 1275 & 1234		α-, β- and γ -phase
NA	NA	β- and γ -phase (NA)

Supplementary Table 3. The melting temperature (T_m) of the initial HP and P&F PVDF films.

Samples	T_m (°C)
180 kg/mol initial HP	164.9/169.4±1
180 kg/mol 1 fold	167.0±1
180 kg/mol 3 fold	166.8±1
180 kg/mol 5 fold	168.9±2
180 kg/mol 7 fold	168.6±1
534 kg/mol initial HP	172.2±1
534 kg/mol 1 fold	171.9±1
534 kg/mol 3 fold	174.0±1
534 kg/mol 5 fold	174.4±2
534 kg/mol 7 fold	173.7±1
670-700 kg/mol initial HP	171.7±1
670-700 kg/mol 1 fold	171.1±1
670-700 kg/mol 3 fold	173.2±2
670-700 kg/mol 5 fold	170.6±2
670-700 kg/mol 7 fold	173.7±1
670-700 kg/mol stretched	169.0±2

T_m: Melting temperature

The folded samples show similar melting points compared to the initial HP materials (no folding) (Supplementary Figure 3).

Supplementary Table 4. The XRD data for initial HP and P&F films. The size of crystallites was calculated using diffraction peaks at about 20.0° and the Scherrer equation. A decrease in the mean size of crystallites is observed after P&F.

Samples	2 Theta corresponding to the reflections of $(110)_{\alpha}$ and/or $(110)/(200)_{\beta}$[°]	FWHM[a)] corresponding to the reflections of (110)α and/or (110)/(200)β [rad]	Mean size of crystallites [nm]
180 kg/mol initial HP	20.1	0.36	22
534 kg/mol initial HP	20.2	0.39	21
670-700 kg/mol initial HP	20.1	0.41	20
180 kg/mol 7 fold	20.8	1.32	6
534 kg/mol 7 fold	20.7	1.87	4
670-700 kg/mol 7 fold	20.6	2.25	3.5
670-700 kg/mol stretched	20.6	0.78	10

a) Full width at half maximum
